# Hospital Meetings, &c.

**Published:** 1899-02-25

**Authors:** 


### HOSPITAL MEETINGS, &C.

### CITY OF LONDON LYING-IN HOSPITAL.

The 148th annual court of governors of the City of London Lying-in Hospital was held at the hospital, City Road, on Wednesday, 15th inst., Mr. Charles Gordon in the chair. The report of the Committee of Management, which was presented and adopted, recorded that during the past year 565 in-patients were admitted—an increase of 44 as compared with last year, and the highest number since 1863—and 1,623 treated as out-patients, a decrease of 45. With regard to the Training School for Midwives and Nurses, there were 47 of the former and 161 of the latter trained, the largest number ever passed through the hospital in one year. In consequence of the increase in the number of pupils the committee propose to enlarge the premises by continuing the present block of buildings to the boundary wall, and so provide a sitting-room and sleeping accommodation for 20 pupils. By this means an additional ward will be at the disposal of the medical staff for the use of patients. The estimated cost of the extension is from £2,000 to £2,500, and to meet this the trustees were authorised to sell £2,000 Consols. During the past year the matron, Miss Fox, has taken over the additional duties of housekeeper, and, though the daily average of inmates was four more than in 1897, expenses under the head of maintenance had been reduced from £3,126 to £2,968—a saving of £157. The ordinary income of the hospital for the year amounted to £4,111, and £675 was received in legacies—a total of £4,786. The ordinary expenditure was £3,383, which, with £165 spent on repairs, improvements, and transfer of stock, totalled £3,549. £1,605 was invested, and including this latter item the accounts show an excess of expenditure over revenue of £368. This, of course, is merely a bookkeeping deficit, more than covered by the credit balance of £605 from the previous year, leaving an actual credit cash balance of £237. General satisfaction was expressed at the state of the hospital's finances, and also at the fact that the sanitary and hygienic condition of the institution remained uniformly good, as was borne out by their having almost a clean sheet as regards maternal deaths, only one having occurred during the year, and that not from septic, but from historic causes. The good work in training nurses and midwives was also referred to, while the Rev. N. J. Devereux, vicar of St. Mary's, Hoxton, bore testimony to the assistance rendered outside the hospital among the poor of Hoxton, Shoreditch, and the neighbourhood. Votes of thanks to the committee, the officers, and to the chairman closed the proceedings.

### CITY OF LONDON HOSPITAL FOR DISEASES OF THE CHEST.

It has been thought desirable by the Committee of Management of the City of London Hospital for Diseases of the Chest to have, in connection with the institution, an association consisting of ladies willing to take an active part in the work of the hospital, and to assist in providing flowers, amusement, and occupation for the patients. A number of well-known ladies, leaders of society and of philanthropy, have interested themselves in the matter, and on Tuesday (14th inst.), at the invitation of the treasurer of the institution, Sir Edward Sassoon, and Lady Sassoon, a reception was held at the hospital, Victoria Park, E., when a most successful inauguration of the Ladies' Association took place. The Lady Rothschild and her daughter, the Hon. Evelina, the Countess of Jersey, the Lady Wolverton, Lady Jeune, Mrs. Bischoffsheim, Mrs. Cator, Mrs. Andrew Motion, Mrs. Thorowgood, Mrs. Heron, Mrs. Vincent Harris, Mrs. Clifford Beale, Mrs. Harrington Sainsbury, Miss Stanley, and many other ladies are giving active support to the movement. A capital entertainment was provided, the Leoni Ladies' Quintette supplying a charming selection of instrumental music, while Messrs. Cecil George, Charles Thomason, J. Lewis Woods, and Arthur Percival were the vocalists. In all, between 200 and 300 guests were present, including Lady Rothschild and many of the ladies mentioned, and other well-known City and West End people whom it was certainly a very good thing to have lured into the far East, which after all is really easily accessible, Cambridge Heath Station being but ten minutes from Liverpool Street, and the hospital itself not more than five minutes' walk away.

### SEAMEN'S HOSPITAL SOCIETY.

Mr. George Lidgett presided at the seventy-eighth annual general meeting of the Seamen's Hospital Society, Greenwich, held at the Institute of Chartered Accountants on Friday last, 17th inst. The chief subject dealt with in the report presented was the important new departure inaugurated during the past year—the establishment of the London School of Tropical Medicine. Early in the year a suggestion was received from the Colonial Secretary that a scheme should be devised by which all newly-appointed medical officers in the Colonial service could receive a sound and systematic training in tropical diseases in this country before taking up their duties in the tropics. Upon the recommendation of a sub-committee appointed to consider the matter, the Committee of Management came to the conclusion that there is in the society's hospitals and dispensaries clinical material for the study of these special forms of disease which cannot be found elsewhere in the country in the same amount and variety, since there are usually under treatment acute cases such as are to be met with in actual practice in the tropics, including Asiatics, Africans, and West Indians, as well as Europeans and natives of the various colonies. Ultimately it was arranged with the Colonial Office that the branch hospital at the docks should be enlarged to a capacity of 45 beds, and that a properly equipped medical school, with laboratory, pathological room, &c., at an estimated cost of £16,000. The Colonial Office will contribute £3,500 towards this, and a further £1,000 per annum, it being arranged that medical officers going abroad in the Colonial service shall go through three or four months' curriculum. The Foreign Office has also arranged for the protectorates under its administration to participate in the benefits and contribute to the funds of the new school. Representatives of both departments will be nominated on the Committee of Management. The school is not, however, to be confined to Government officials, but will be thrown open to the great missionary societies and other corporations, whose medical officers may thus go through a course of study in their special duties before proceeding abroad. That the ordinary work of the society was carried on with, if anything, increased activity during the year is shown by the fact that 2,548 in-patients and 22,850 out-patients were dealt with at the various establishments, the total—25,398—being even in excess of last year's exceptionally large number. The financial statement is not quite so satisfactory, the total ordinary income amounting only to £12,711, as compared with £14,967 last year; while the total ordinary expenditure was £17,477. Legacies amounted to £3,026, and expenditure on building and repairs to £1,299.

The report was adopted without discussion, and the meeting ended with the customary votes of thanks.

